# Marriages, Births and Deaths

**Published:** 1903-08-29

**Authors:** 


### The Hospital.

A JOURNAL OF

The Medical Sciences and Hospital Administration.

VOL. XXXIV.—No. 883. AUGUST 29, 1903.

### MARRIAGES, BIRTHS AND DEATHS.

THE Quarterly Return of Marriages, Births and Deaths, issued by the Registrar-General, may perhaps be described as the most remarkable known example of the conveyance of useful knowledge in a curiously unattractive form. When the late Dr. Farr held office as Superintendent Registrar, it was his custom, possibly from a sense of this unattractiveness and from a desire to diminish it, to adorn the preliminary pages of his returns with highly interesting disquisitions, always exceedingly well worthy of perusal and often worthy of study, but which might have been held to commit the department to views possibly entertained only by the distinguished writer. Dr. Farr's successors have departed from this custom, and have probably exercised a sound discretion in doing so; but the comparatively barren paragraphs in which they summarise the chief results of the tabulations of each successive quarter have done little to lighten the labours of those whose duty it is to extract from them such lessons as they may be calculated to convey. That there are such lessons, and that they are, in many cases, of supreme importance to the community, it is scarcely possible to doubt.

For some reason which is not apparent upon the surface, and of which we have never heard any satisfactory explanation, the returns of marriages are always three months in arrear of the returns of births and deaths, so that the quarterly pamphlet just issued brings down the former only to March 31st, but the two latter to June 30th. The annual number of marriages in the first quarter was at the rate of 11·1 persons married to every thousand of the estimated population, as against an average rate of 11·5 per thousand in the first quarters of the 10 years 1893–1902.

The diminution in the number of births is, we regret to observe, even greater than that in the number of marriages, the total being 241,778, or in the proportion of 29·1 annually per thousand of the population, the mean rate in the 10 preceding second quarters having been 29·7. The difference between 0·1 and 0·7 does not at first sight appeal strongly to the imagination, nor does it seem to point to any probability of the speedy depopulation of the country; but, nevertheless, it means, in round numbers, that the children born during the quarter were fewer than the average by more than 20,000.

To the medical profession, of course, the most interesting element in the return is the death-rate, or the figures which tell of comparative success or failure in our contest with the many enemies by which human life is continually beset. On this side of the question it is pleasant to have to record an improvement which for some time has been continuous. The deaths of the quarter were scarcely more than half of the number of births, and were at the rate of only 14·9 annually per thousand persons living, as against 16·6 as the average of the second quarters of the ten preceding years. A still more important change in the same direction, because it affords one of the most delicate tests of the sanitary state of the community, is the diminished mortality among children under one year of age, a mortality which has fallen to 110 per thousand births, as against 124 per thousand for the second quarters of the ten preceding years. Figures are now furnished, moreover, by the great majority of local health authorities, which show the number of cases of infectious disease notified within their respective boundaries, and which, when compared with the deaths registered from the same causes, enable us to ascertain the percentage mortality from each notifiable disease within definite limits. Thus, in London, with its population of 4,613,812, there were notified 162 cases of small-pox with 2 deaths; and 3,151 of scarlet fever with 82 deaths. In Leicester, a comparatively unvaccinated locality, there were 195 cases of small-pox with 14 deaths, and 129 cases of scarlet fever with 4 deaths. The Leicester death-rate in occurring cases of scarlet fever, it will be observed, was only a fraction higher than that of London, while the similar death-rate from small-pox was 1 in 14, as compared with 1 in 81 in London. Other instructive figures of a like kind might be cited did space allow; and it is manifest that a continued comparison between the prevalence and the fatality of the same disease in different localities will soon become a matter of the first importance to sanitary reformers. It is the more to be regretted that there are still four local authorities which do not sanction the transmission of their notifications of disease. They are in the worst sense obstructives; and it is fitting that their names should be placed on record, much as a gamekeeper nails vermin to the door of a burn. The defaulters are Aldershot, Ramsgate, Cheltenham, and Blackpool; and we can only hope that they may soon desert the policy of the ostrich, and may join with others in the supply of information which is of high importance for the safety of the public.

